# Correlation of occupational stress with depression, anxiety, and sleep in Korean dentists: cross-sectional study

**DOI:** 10.1186/s12888-017-1568-8

**Published:** 2017-12-12

**Authors:** Kyung-Won Song, Won-Seok Choi, Hee-Jung Jee, Chi-Sung Yuh, Yong-Ku Kim, Leen Kim, Heon-Jeong Lee, Chul-Hyun Cho

**Affiliations:** 1 Department of Oral Medicine, School of Dentistry, Dankook University, Cheonan, South Korea; 2 Department of Psychiatry, Korea University College of Medicine, Seoul, South Korea; 3 Department of Biostatistics, Korea University College of Medicine, Seoul, South Korea; 4 Department of Prosthodontics, College of Dentistry, Yonsei University, Seoul, South Korea

**Keywords:** Occupational stress, Sleep, Depression, Anxiety, Dentist, Mental health

## Abstract

**Background:**

This study aimed to investigate the degree of occupational stress and the clinical mental state of dentists. In addition, we investigated the correlation of occupational stress with depression, anxiety, and sleep among dentists in Korea.

**Methods:**

A cross-sectional survey on 231 dentists was conducted using the Doctor Job Stress Scale, Center for Epidemiologic Studies Depression Scale (CES-D), State-Trait Anxiety Index (STAI), and Pittsburgh Sleep Quality Index (PSQI). Correlation of occupational stress with mental health was investigated by adjusted multiple regression analysis.

**Results:**

The scores of CES-D, STAI, and PSQI revealed a significant correlation with the Doctor Job Stress Scale (*t* = 3.93, *P* < 0.0001; *t* = 4.05, *P* < 0.0001; *t* = 4.18, *P* < 0.0001, respectively). In particular, patient factors and clinical responsibility/judgment factors were significantly associated with depression (*t* = 2.80, *P* = 0.0056; *t* = 4.93, *P* < 0.0001, respectively), anxiety (*t* = 2.35, *P* = 0.0195; *t* = 5.11, *P* < 0.0001, respectively), and sleep (*t* = 3.78, *P* = 0.0002; *t* = 4.30, *P* < 0.0001, respectively), whereas work factors were not associated with any mental health state.

**Conclusions:**

This study confirms that dentists as professions experience more severe mental states. For successful mental health care among dentists, stress management focusing on interpersonal relationship with patients and responsibility as an expert rather than the intensity of work should be considered.

## Background

Stress is a state of human psychological conflict arising from external threats that are constantly above or beyond the ability to manage, and an indispensable survival factor for individuals with limited resources in modern life [1]. Work-related stress promotes a successful role, and a certain degree of stress is a natural phenomenon experienced by humans and is a part of their survival and well-being. However, excessive chronic stress could adversely affect the physical and mental health states [2, 3].

Occupational stress negatively affects the quality of life and health, resulting in social and economic costs [4, 5]. The relationship between occupational stress and depression has been also previously reported [6–8]. Given that depression is closely associated with suicide, the relationship between occupational stress and suicide needs to be considered. Indeed, several studies have reported that occupational stress, such as excessive workload or working time, was closely related to suicide [9–11].

Professional occupational stress is high, particularly in physicians due to requirements such as high vocational consciousness and moral standards, and medical knowledge and skills through hard training [12, 13]. Physicians are recognized as social authorities because of their expertise. However, they are exposed to high levels of stress because of excessive workloads, relationships with patients, responsibility for the lives and health of others, and urgency of the work itself [14, 15]. When physicians experience high levels of stress, their productivity and quality of work deteriorate, their relationships to patients worsens, and they develop deleterious behaviors such as excessive smoking or heavy alcohol drinking [16, 17]. Occupational stress among physicians not only could affect patients adversely but also could ultimately decrease the overall quality of health care service [18, 19]. Moreover, some reported that the higher the occupational stress, the higher the incidence of suicide particularly in female physicians [20–22].

Dentists are also known to experience more occupational stress than other professions [19], owing presumably to the working environment. Dentists should maintain the same posture for a long time with the waist being bent [23]. The surgical workplace is limited to a narrow space called the oral cavity. The noise perceived during the procedures and the smell of the disinfectants and materials used during treatment stimulate the senses and affect the dentists’ mental health [3]. Dentists inhale mercury vapors during amalgam treatment [24], which may cause mercury poisoning, leading to depression, irritability, and insomnia [25, 26]. Dentists are typically perfectionists who are easily frustrated when not reaching the ideal therapeutic goal. The burden to meet the patients’ high aesthetic demands and the persistent desire for technical perfection are also major stress factors [25, 26]. A considerable number of dentists have been obsessive and compulsive since they were dental college students. Dental college students need social awareness and achieve high social status, but they have unrealistic expectations and unnecessarily over-demanding behavioral patterns [1–3]. The level of stress they need to deal with during their undergraduate studies is very high and increases with graduation. According to one study, 47% of second-year students and 67% of final-year students in dental school had anxiety, while 15% of second-year students and 14% of final year students had pathologic depression [16]. Mental health problems including anxiety, depression, and suicide are serious problems to watch out for in dentists [2, 3].

Dentist’s occupational stress share many similarities with doctor’s occupational stress, but until now there has been a lack of research on dentist’s occupational stress and mental health state in Korea. Therefore, it would be meaningful to investigate the relationship between the dentist’s occupational stress and mental health states which are mood, anxiety, and sleep. The purpose of our study is to investigate the degree of occupational and psychosocial stress, and the clinical mental state of depression, anxiety, and sleep in dentists with subjective questionnaires. In addition, we find the correlation between the occupational stress and each mental health states with adjustment of other socio-demographical variables which could affect the clinical states as confounding variables. Furthermore, we sought to find out which of the occupational stress factors are closely related to mental health among dentists.

## Methods

### Study population

The present study was performed from September to October 2016, and the actual assessment through the questionnaire evaluation of dentists was conducted from September to November 2016. Only licensed dentists were included in this study. After obtaining the contact information of about 1520 dentists from several regional societies of the Korean Dental Association, the Korean Academy of prosthodontics, and the Korean academy of orofacial pain and oral medicine, we set twice a short message service to guide the online survey address (ko.surveymonkey.com). All the participants provided informed consent before responding to the questionnaire evaluation of this study via an online survey. Study protocols were ethically approved by the Institutional Review Board of Dankook University and were conducted in accordance with the Declaration of Helsinki.

At the end of the survey, a total of 283 participants completed the questionnaire. Of these, 52 participants were excluded from the analysis because they did not complete the responses to all the questionnaires or there were errors in the responded data. Finally, the responses of 231 dentists were included in the final analysis.

### Assessment

#### Assessment of socio-demographic and general information

The socio-demographic and general health information of the participants was confirmed. The following information was recorded: sex, age, religion, social status (upper, middle, lower), and income (generally high, middle, and lower). The marital status was divided into single, married, or living together, divorce, separation, bereavement, and others. The participants were asked about the status of alcohol consumption (not drinking, less than 3 days/month, 1–2 days/week, and >3 days/week) and smoking (current and past smoking status). To assess the occupational state of the dentists, current job (private outpatient clinic practitioner, university hospital professor, university hospital resident, hospital employed dentist, clinic employed dentist, and others), average working hours per day during weekdays (4–8 h, 8–10 h, > 10 h, and others), and job satisfaction (satisfied, not bad, and unsatisfied) were also recorded. The respondents were additionally asked about the prospects for future status of dentists (expect to be higher than current, to remain the same as current, and to fall below than current level).

#### Assessment of occupational stress

The degree of stress was measured using the abbreviated Doctor Job Stress Scale [27]. Three factors related to the Doctor Job Stress presented by Kang et al. were categorized into work factors, patient factors, and clinical responsibility/judgment factors. In total, three questionnaires were extracted for each factor. Among the Doctor Job Stress items, work factors were assessed through the statements “I should treat too many patients in a short time”, “I am usually tired after finishing daily work,” and “I can’t have much time with family because of work.” Patient factors were assessed through the following statement: “Dealing with patients who are not compliant” and “Treating patients whose prognosis on not good regardless of intensive care”. The clinical responsibility/judgment factors were evaluated using the following statements: “Having a pressure to learn advanced medical knowledge and skill”, “Worrying about medical malpractice and medical dispute,” and “Making a decision which affect serious impact on patient’s condition” [27].

#### Assessment of psychosocial stress

The degree of psychosocial stress was measured using the Brief Encounter Psychosocial Instrument Korean version (BEPSI-K). BEPSI-K was developed to evaluate the negative effects of stress on health based on a stress model as a dynamic interaction; its reliability and validity were verified [28, 29]. BEPSI-K consists of five questionnaires that assess the psychological states of the past month. Each questionnaire item is assessed on a 5-point Likert scale ranging from ‘not at all’ to ‘always present’. The BEPSI-K score uses the mean value of all questionnaires, and the psychosocial stress group was indicated by a score ≥ 2.4 [29].

#### Assessment of mental health state (depression, anxiety, and sleep)

In this study, we hypothesized that job stress of dentists would affect mood, anxiety, and sleep. Therefore, we assessed dentists’ mental health state (mood, anxiety and sleep) through subjective questionnaires.

The Center for Epidemiologic Studies Depression Scale (CES-D) was applied to evaluate the severity of depression. CES-D is a 20-item questionnaire with a 5-point scale [30]. The CES-D Korean version demonstrated a high reliability [31]. In the present study, we defined *probable depression* as a CES-D score ≥ 16, and *definite depression* as a CES-D score ≥ 25 [31].

The State-Trait Anxiety Index (STAI) was applied to evaluate the severity of anxiety [32]. It is a 20-item self-report developed to assess the level of situation-related state anxiety (STAI-S) and trait anxiety (STAI-T). The Korean version of STAI was also validated [33]. We used only STAI-S in the present study, and defined *mild anxiety* as a STAI-S score ≥ 54 and *severe anxiety* as a STAI-S score ≥ 64 [33].

The self-rating Pittsburgh Sleep Quality Index (PSQI) was applied to evaluate the sleep quality during the last month. It is consisted of 19 individual items that generate seven components (range 0–3) on subjective sleep quality, sleep latency, sleep duration, habitual sleep efficiency, sleep disturbances, use of sleeping medication, and daytime dysfunction. The sum of the seven components yields one sleep quality score (range 0–21). A global PSQI score greater than 5 indicates poor sleep quality [34]. The PSQI has been translated into Korean version and validated [35].

### Statistical analysis

The differences in Doctor Job Stress, psychosocial stress, sleep quality, and degree of depression and anxiety according to the respondents’ sociodemographic and job-related characteristics were examined by an independent sample t-test and one-way analysis of variance (ANOVA). In addition, Doctor Job Stress items were categorized into three factors: work factors, patient factors, and clinical responsibility/judgment factors. The relationship between each factor based on the Doctor Job Stress Scale with depression, anxiety, and sleep was examined by multiple regression analysis. When the t-test and ANOVA were performed, we considered variables with significance of *P* < 0.1 as possible confounding variables. Therefore, multiple regression analysis was performed with adjusting these variables. For adjusting the age as possible confounding variable, we performed regression analysis of age with Doctor Job Stress Scale with each three factors, BEPSI-K, CES-D, STAI-S, and PSQI; we considered the significance of P < 0.1 as confounding factor with adjusting age for multiple regression analysis. All statistical analyses were performed using SAS (version 9.4; SAS Institute Inc., Cary, NC, USA). The significance level α was set at 0.05.

## Results

Of the 231 dentists included in this study, there were 74 female participants (32.0%), and the mean age (± standard deviation [SD]) was 41.77 ± 9.79 years (range 24–64 years). Out of the included participants, 153 (66.2%) were private outpatient clinic practitioners, 30 (13.0%) were employed at the clinic, 15 (6.5%) were employed at a hospital, 13 (5.6%) were residents, and 4 (1.7%) were professors, and 16 (7.0%) were categorized as others. Other sociodemographic characteristics of the investigated dentists were listed in Table 1.

**Table 1:** Sociodemographic characteristics and general information of the participants (*N* = 231)

Variables	Number	Percent
Sex		
Male	157	68.0
Female	74	32.0
Socioeconomic status		
High	59	25.5
Middle	167	72.3
Low	5	2.2
Exercise frequency		
No exercise	60	26.0
1–2 times per week	112	48.5
3–4 times per week	51	22.0
> 5 times per week	8	3.5
Alcohol consumption		
None	73	31.6
< 3 times per month	70	30.3
1–2 times per week	65	28.1
> 3 times per week	23	10.0
Smoking status		
Smoking	39	16.9
No smoking	192	83.1
Marital status		
Unmarried	51	22.1
Married	174	75.3
Divorced, Separated, Bereaved	6	2.6
Occupation		
Private outpatient clinic practitioner	153	66.2
Professor	4	1.7
Resident	13	5.6
Hospital employed dentist	15	6.5
Clinic employed dentist	30	13.0
Other	16	7.0
Working time per day		
4–8 h	19	8.2
8–10 h	83	36.0
> 10 h	128	55.4
Other	1	0.4
Job satisfaction		
Satisfied	81	35.0
Not bad	127	55.0
Unsatisfied	23	10.0

The mean score (± SD) of Doctor Job Stress Scale was 3.23 ± 0.54. Among the Doctor Job Stress Scale, mean score of work factors, patient factors, and clinical responsibility/judgment factors were 3.30 ± 0.78, 3.03 ± 0.66, and 3.34 ± 0.70, respectively. The mean score (± SD) of BEPSI-K, CES-D, STAI-S and PSQI were 2.04 ± 0.57, 15.58 ± 9.69, 43.06 ± 10.07 and 5.26 ± 2.96, respectively. The number of probable depression group was 57 (24.7% of total dentists) and definite depression group was 44 (19.0% of total dentists), and the number of problematic depression was 101 (43.7%). The clinical (problematic) state of psychosocial stress, depression, anxiety, and sleep according to the scores of BEPSI-K, CES-D, STAI-S, and PSQI were listed in Table 2.

**Table 2:** Clinical state of psychosocial stress, depression, anxiety, and sleep according to the scores of BEPSI-K, CES-D, STAI-S, and PSQI

	BEPSI-K (cut-off score: 2.4)	CES-D (cut-off score: 16)	STAI-S (cut-off score: 54)	PSQI (cut-off score: 5)
Mean ± SD	2.04 ± 0.57	15.58 ± 9.69	43.06 ± 10.07	5.26 ± 2.96
	Psychosocial stress (N, %)	Depression (N, %)	Anxiety (N, %)	Sleep (N, %)
Clinical state				
Problematic (≥ cut-off score)	127 (55.0%)	101 (43.7%)	30 (13.0%)	127 (55.0%)
Normal (< cut-off score)	104 (45.0%)	130 (56.3%)	201 (87.0%)	104 (45.0%)

BEPSI-K, Brief Encounter Psychosocial Instrument Korean version; CES-D, Center for Epidemiologic Studies Depression Scale; N, number of participants; PSQI, Pittsburgh Sleep Quality Index; SD, standard deviation; STAI-S, State-Trait Anxiety Index situation-related state anxiety

Table 3 summarizes the differences in Doctor Job Stress Scale, BEPSI-K, CES-D, STAI-S, and PSQI according to the respondents’ sociodemographic and job-related characteristics. In the low-income group, psychosocial stress was higher than in the middle-income and high-income groups (*P* = 0.0004). A lower sleep quality was observed in the low-income group compared with the middle- and high-income groups, although not statistically significant (*P* = 0.0655). Depression and state anxiety were more severe in the low social status and low income groups (*P* < 0.0001). Alcohol consumption was related to sleep (*P* = 0.0187), depression (*P* = 0.0061), and anxiety (*P* = 0.0003). The groups with a high alcohol consumption, particularly those drinking more than once a week showed lower quality of sleep (*P* = 0.0187), and more severe depression (*P* = 0.0061) and state anxiety (*P* = 0.0003). The marital status was associated with the Doctor Job Stress Scale score. In divorced, separated, and bereaved groups, a higher Doctor Job Stress score was observed than in those of the married and unmarried groups (*P* = 0.0254). The working time per day was also related to Doctor Job Stress score (*P* = 0.0024), psychosocial stress (*P* = 0.0005), depression (*P* = 0.0010), and anxiety (*P* < 0.0001). Higher average daily working hours were associated with higher Doctor Job Stress and psychosocial stress scores, and more severe depression and state anxiety, but not with the quality of sleep (*P* = 0.2277). Job satisfaction was associated Doctor Job Stress, psychosocial stress, sleep, depression, and anxiety (all, *P* = 0.0001). The high job-satisfaction group showed lower Doctor Job Stress score and psychosocial stress, while the unsatisfied group showed a lower sleep quality and more severe depression and state anxiety. Sex, frequency of exercise, smoking status, and future prospects were not associated with the Doctor Job Stress score, psychosocial stress, sleep quality, depression, and state anxiety.

**Table 3:** The differences in the Doctor Job Stress Scale, psychosocial stress (BEPSI-K), sleep quality (PSQI), depression (CES-D), and anxiety scale (STAI-S) according to Sociodemographic characteristics

Variables	Doctor job stress			Psychosocial stress (BEPSI-K)			Sleep quality (PSQI)			Depression scale (CES-D)			Anxiety scale (STAI-S)		
	Mean	SD	*p*-value	Mean	SD	p-value	Mean	SD	*p*-value	Mean	SD	*p*-value	Mean	SD	*p*-value
Sex			0.4637			0.4004			0.5297			0.9048			0.9129
Male	3.24	0.51		2.04	0.56		5.34	2.89		15.64	9.81		43.01	10.20	
Female	3.19	0.59		2.02	0.59		5.08	3.11		15.47	9.51		43.16	9.84	
Socioeconomic status			0.2882			*0.0018* ^†*^			0.3047			< *0.0001* ^†*^			< *0.0001* ^†*^
High	3.26	0.58		1.86	0.52		4.85	2.64		11.69	9.12		38.49	8.24	
Middle	3.21	0.53		2.08	0.56		5.37	3.03		16.59	9.20		44.22	9.78	
Low	3.44	0.21		2.64	0.62		6.60	3.91		27.80	15.24		58.20	15.01	
Income			0.3765			*0.0004* ^†*^			0.0655^*^			< *0.0001* ^†*^			<* 0.0001* ^†*^
High	3.20	0.51		1.88	0.53		5.03	2.86		12.25	8.69		39.89	8.40	
Middle	3.21	0.57		2.05	0.56		5.07	2.93		16.28	9.73		43.46	10.31	
Low	3.33	0.49		2.31	0.56		6.25	3.11		20.33	9.33		48.28	10.29	
Exercise frequency			0.9608			0.8905			0.6094			0.4523			0.6533
No exercise	3.24	0.52		3.20	0.59		4.82	2.51		15.62	9.00		43.67	9.27	
1–2 times/week	3.23	0.53		3.28	0.62		5.43	2.80		15.63	9.70		43.20	9.88	
3–4 times/week	3.21	0.61		3.25	0.59		5.37	3.63		14.65	9.96		41.67	11.13	
≥ 5 times/week	3.14	0.36		3.23	0.59		5.50	3.70		20.63	13.03		45.38	12.22	
Alcohol drinking			0.5327			0.3609			*0.0187* ^†*^			*0.0061* ^†*^			*0.0003* ^†*^
None	3.21	0.53		3.20	0.61		4.75	3.08		14.26	10.52		41.40	10.50	
≤ 3/month	3.18	0.59		3.19	0.59		4.81	2.66		13.44	7.28		40.33	8.07	
1–2 times/week	3.24	0.50		3.34	0.58		5.97	2.92		18.23	9.36		46.57	9.97	
≥ 3 times/week	3.37	0.49		3.36	0.61		6.22	3.07		18.83	12.11		46.70	11.05	
Smoking status			*0.0229* ^†*^			0.8549			0.1947			0.5014			0.3956
Smoking	3.36	0.37		3.24	0.51		5.82	2.54		16.54	9.84		44.31	9.18	
No smoking	3.20	0.56		3.25	0.62		5.15	3.03		15.39	9.68		42.80	10.24	
Marital status			*0.0254* ^†*^			0.5241			0.7798			0.1007			*0.0069* ^†*^
Unmarried	3.14	0.61		3.24	0.68		5.48	2.79		18.04	9.40		46.66	10.38	
Married	3.23	0.51		3.24	0.58		5.16	3.05		14.70	9.79		41.78	9.76	
Divorced, Separated, Bereaved	3.83	0.46		3.61	0.43		6.17	1.47		20.50	4.85		48.50	8.31	
Other	3.56			3.33	.		6.00	.		17.00	.		53.00		
Occupational status			*0.0156* ^†*^			0.2370			0.8883			0.1544			0.0929^*^
Private outpatient clinic practitioner	3.20	0.51		3.25	0.57		5.20	2.98		14.68	9.71		41.86	9.69	
Professor	3.44	0.58		3.58	0.40		6.50	2.38		21.75	11.87		46.75	8.18	
Resident	3.45	0.48		3.21	0.64		5.77	2.35		17.92	7.01		46.00	7.12	
Hospital employed dentist	3.41	0.53		3.43	0.65		5.43	2.61		18.30	9.66		47.37	10.94	
Clinic employed dentist	3.21	0.59		3.09	0.56		4.73	3.15		13.13	6.06		43.60	6.41	
Other	2.88	0.65		3.05	0.73		5.25	3.91		18.00	12.35		42.56	14.63	
Working time per day			*0.0024* ^†*^			*0.0005* ^†*^			0.2277			*0.0010* ^†*^			< *0.0001* ^†*^
4–8 h	3.07	0.61		3.10	0.62		5.27	3.32		14.12	9.98		40.08	10.53	
8–10 h	3.28	0.46		3.35	0.55		5.15	2.73		15.91	9.19		44.18	9.39	
≥ 10 h	3.51	0.52		3.17	0.61		5.68	2.58		17.95	8.28		46.63	6.07	
Other	3.67			5.00	.		11.00	.		50.00	.		78.00		
Job satisfaction			*0.0001* ^†*^			< *0.0001* ^†*^			*0.0001* ^†*^			< *0.0001* ^†*^			< *0.0001* ^†*^
Satisfied	3.12	0.56		2.91	0.52		4.25	2.48		10.06	6.91		37.15	7.95	
Not bad	3.22	0.49		3.36	0.53		5.64	3.16		17.28	9.30		45.07	9.51	
Unsatisfied	3.65	0.54		3.84	0.54		6.74	2.28		25.65	8.83		52.74	7.81	

BEPSI-K, Brief Encounter Psychosocial Instrument Korean version; CES-D, Center for Epidemiologic Studies Depression Scale; N, number of participants; PSQI, Pittsburgh Sleep Quality Index; SD, standard deviation; STAI-S, State-Trait Anxiety Index situation-related state anxiety

^†^; *p*-value <0.05

^*^; *p*-value <0.1 (confounding factors for multiple regression analysis)

The statistical analysis was performed by an independent sample t-test or one-way analysis of variance (ANOVA)

Table 4 summarizes the results of multiple regression analysis with adjustment. As mentioned above, we considered variables with a *P* < 0.1 as possible confounding variables; the confounding variables among Table 3 were adjusted for multiple regression. After searching for covariance of age by regression analysis, we found a significance of P < 0.1 with STAI-S (*P* = 0.027) and clinical responsibility/judgment factor from the Doctor Job Stress Scale (*P* = 0.086). Therefore, we adjusted age as a confounding factor for multiple regression analysis of STAI-S and clinical responsibility/judgment factor from the Doctor Job Stress Scale. In the adjusted multiple regression analysis, CES-D, STAI-S, and PSQI revealed a significant correlation with the Doctor Job Stress Scale and BEPSI-K (Doctor Job Stress Scale: B = 4.42, *t* = 3.93, *P* < 0.0001; B = 4.52, *t* = 4.05, P < 0.0001; B = 1.61, *t* = 4.18, P < 0.0001, respectively; BEPSI-K: B = 8.84, *t* = 9.20, P < 0.0001; B = 8.26, *t* = 8.43, P < 0.0001, B = 2.55, *t* = 7.26, P < 0.0001, respectively). Among the Doctor Job Stress Scale factors, the patient and clinical responsibility/judgment factors were significantly associated with depression (B = 0.81, *t* = 2.80, *P* = 0.0056 and B = 1.32, *t* = 4.93, P < 0.0001, respectively), anxiety (B = 0.71, *t* = 2.35, *P* = 0.0195 and B = 1.35, *t* = 5.11, P < 0.0001, respectively), and sleep (B = 0.37, *t* = 3.78, *P* = 0.0002 and B = 0.40, *t* = 4.30, P < 0.0001, respectively). In contrast, the work factors were not associated with any mental health state.

**Table 4:** Multiple regression analysis after adjusting confounding variables

	Regression coefficient (B)	Standard Error	t Value	*P*-value
*CES-D*				
BEPSI-K	8.84	0.96	9.20	< *0.0001* ^**†**^
Doctor job stress scale	4.42	1.12	3.93	< *0.0001* ^**†**^
Work factors	0.37	0.28	1.31	0.1928
Patient factors	0.81	0.29	2.80	*0.0056* ^**†**^
Clinical responsibility/judgment factors	1.32	0.27	4.93	< *0.0001* ^**†**^
*STAI-S*				
BEPSI-K	8.26	0.98	8.43	< *0.0001* ^**†**^
Doctor job stress scale	4.52	1.11	4.05	< *0.0001* ^**†**^
Work factors	0.42	0.28	1.52	0.1290
Patient factors	0.71	0.30	2.35	*0.0195* ^**†**^
Clinical responsibility/judgment factors	1.35	0.26	5.11	< *0.0001* ^**†**^
*PSQI*				
BEPSI-K	2.55	0.35	7.26	< *0.0001* ^**†**^
Doctor job stress scale	1.61	0.38	4.18	< *0.0001* ^**†**^
Work factors	0.14	0.10	1.48	0.1414
Patient factors	0.37	0.10	3.78	*0.0002* ^**†**^
Clinical responsibility/judgment factors	0.40	0.09	4.30	< *0.0001* ^**†**^

BEPSI-K, Brief Encounter Psychosocial Instrument Korean version; CES-D, Center for Epidemiologic Studies Depression Scale; PSQI, Pittsburgh Sleep Quality Index; STAI-S, State-Trait Anxiety Index situation-related state anxiety

^†^; *p*-value <0.05

## Discussion

The mean score of the Doctor Job Stress Scale was 3.23 ± 0.54, which is consistent with the previously reported score of 3.30 in Korean practitioners and employed doctors. Furthermore, when the Doctor Job Stress Scale was divided into three factors, the mean scores were not significantly different [27]. The average BEPSI-K score of 2.04 was also higher than that of the general population (1.72) [36] and the first-visited patients who visited a familial medicine clinic (1.87) [29]. The psychosocial stress in dentists was higher than the general population in the present study, which is not consistent with the lower score of 2.19 that was previously reported [15].

In our study, dentists tended to experience more common or severe difficulties related to depression, anxiety, and sleep that the general population. In the case of CES-D, the probable depression group corresponded to 24.7% of the total surveyed dentists compared with 23.1% men and 27.4% women reported in a previous study on the general population [31]. No remarkable differences in depression according to the CES-D was noted between the dentists and general population, but the definite depression group (19.0% of the total surveyed dentists) scored higher than those of men (estimated incidence of 6.8%) and women (estimated incidence of 10.4%) in the general population [31]. A study performed in Korea reported a PSQI average score of 4.06 ± 2.08 in the general population [35]. Another study on 4634 train drivers reported a mean PSQI score of 3.50 ± 2.45 (range 0–19), and 792 train drivers (17.0%) reported sleep problems (PSQI ≥5) [37]. The dentists included in the present study (mean PSQI, 5.26 ± 2.96, with 55.0% of dentists with PSQI >5) scored worse on the subjective quality of sleep questionnaire than the general population and train drivers. In the case of STAI-S, a mean of 43.01 ± 10.20 in men and 43.16 ± 9.84 in women was observed, which is consistent with previously reported average scores of 40.91 ± 9.84 in men (*N* = 102) and 42.20 ± 9.06 in women (*N* = 85) [38]. These results were higher than previous results from a study in 298 healthy controls with type D personality (mean STAI-S, 36.5 ± 26.3) and 656 healthy controls without type D personality (mean STAI-S 26.3, ± 8.0) [39]. Our results indicate that dentists were more likely to have higher state anxiety than the general population.

From the results of our multiple regression analysis, only the patient and the clinical responsibility/judgment factors of the Doctor Job Stress Scale were significantly related to depression, anxiety, and sleep. This suggests that the burden of acquiring new medical knowledge, and the fear of medical accidents and conflicts with the patients could be the major stressors leading to depression, anxiety, and sleep problems in dentists. Furthermore, work factors arising from occupational stress were not significantly correlated with the mental health states (depression, anxiety, and sleep problems). The burnout could not be the result of the workload since the affected dental practitioner may treat only a certain number of patients at the scheduled time. Therefore, the patient and clinical responsibility/judgment factors might significantly affect mental health state, but not work factors. Through changing the dental treatment model from disease-centered to patient-centered, the patient’s requirements have increased together with the doctor’s responsibilities, and the relationship and communication between doctor and patient have become increasingly important [40, 41]. This may have additionally increased the risk of burnout in dentists [42, 43]. Moreover, most of the dental procedures are surgical practices, with irreversible outcomes, thus increasing the pressure of the patients’ expectations. This may justify the association between clinical responsibility/decision factors and overall mental health. Indeed, it has been found that not only dentists but also oral and maxillofacial surgeons have a high burnout risk [44].

Previous studies on occupational stress and mental health in dentists have identified burnout as a predisposing factor of depression [45, 46]. Burnout indicates mental or emotional exhaustion owing to the long-term exposure to stress [47]. When dentists suffer from burnout, they typically underestimate their accomplishments in a negative and cynical manner in front of their patients. In the burnout state, the stress adaptation mechanism does not function properly and does not recover to the normal state, resulting in increased job turnover and absenteeism, lack of job commitment, and job dissatisfaction [48–51]. In this study, burnout was indirectly examined through occupational stress (Doctor Job Stress Scale) and psychosocial stress (BEPSI-K). Our findings indicated that burnout due to occupational stress may be associated with mental health (depression, anxiety, and sleep problems) in dentists.

Dentists are exposed to a high risk of anxiety and depression since their training as dental college students [16, 25, 47, 52]. They are also exposed to a high level of stress related to the number of patients per day and workload, general financial status, desire for patient’s excessive requirements, technological perfection needs, and fear of litigation and making mistakes [2, 16, 17]. In a study with more than 3500 dentists, 34% reported physical or emotional exhaustion, 38% reported constant or frequent worries or anxieties, and 26% reported headaches and/or backaches [53]. However, not many dentists are able to relieve their stress properly. According to one study, only 10% of the dentists reported having time to relax and only 6% said they had a hobby. Moreover, 24% of the dentists reported not having any activities, while the majority stated having passive stress coping skills [54]. Although our study did not investigate these factors, the dentists included in this study stated that they were particularly stressed due to the interpersonal relationship with the patient, clinical responsibility, and pressure to make important decisions, which all were closely related to their mental states. It will be necessary to address proper stress management to dental college students and dentists and to provide counseling services to dentists to prevent mental health problems caused by occupational stress and burnout.

In the United States, only 27 of 54 dental schools offered lectures on occupational stress with an average lecture time 4.15 h [24] In Korea, such comparisons are not possible because the dental curriculum related to stress management is yet to be investigated. Nevertheless, stress management in Korea is considered to be neglected compared with the United States, and a systematic education curriculum should be prepared in the future for dentist-tailored stress management. The California Dental Association established a hotline service to provide confidential counseling to dentists who are suffering from alcohol and drug addiction and mental illness. Similarly, the necessary support has been provided to prevent or treat job stress related mental problems in Canadian Dental Association, the United Kingdom General Dental Council, and the Minnesota Dental Association [2, 26]. A systematic approach will be needed to manage and support stress and mental health among dentists worldwide, including Korea. Practical help should be provided in a wide range of individuals ranging from dental college students at the time of learning to professional dentists who are actively practicing.

In this study, the high job-satisfaction groups displayed lower job stress, depression, and anxiety, and better sleep quality. Future studies should focus on ways to increase job satisfaction in dentists. In recent studies, intrinsic motivating factors (e.g., occupational calling) have been found to be more closely related to the physicians’ well-being and burnout than extrinsic motivating factors (e.g., annual income) [55, 56]. Therefore, further research and practical applications to improve intrinsic motivating factors should be considered, as they seem to pose an essential challenge for the mental health of dentists.

There are several limitations to our present study. First, this study adopted a cross-sectional design with subjective scales for the assessment of stress and mental health. In a cross-sectional study, causal relationships cannot be identified by multiple regression analysis alone. However, we examined various sociodemographic variables and evaluated occupational and psychological stress as well as mental health states using proven scales. Moreover, we improved the reliability of the results by identifying and adjusting the confounding variables through sophisticated statistical analysis. In order to clarify the causal relationships, it will be necessary to carry out additional prospective research. Second, we did not investigate the period of cumulative clinical experience. In previous studies, as the clinical career builds up, it has been reported that the dentists have improved the skills to manage and cope with stress, thereby reducing occupational stress [2, 3]. If additional clinical careers were examined, it would be possible to investigate whether the reduction of occupational stress and the degree of sleep quality, depression, and anxiety are associated according to period of cumulative clinical experience. We explored the age as covariate for multiple regression analysis, and adjusted age as a confounding factor in several factors. Third, the sample size was relatively small. In order to increase the statistical power, it is essential to include a much larger sample size. However, this study was conducted with professional dentists, and when considering the proportion of dentist in the general population, the sample size of this study seems to be sufficient to analyze and conclusively interpret the results.

## Conclusion

We performed a comprehensive study of sociodemographic, occupational and psychosocial stress, and mental health states in dentists who are specialized profession, with reasonable and detailed analysis of various variables. Finally, this study revealed that overall occupational stress was related to mental health state in the dentists, especially occupational stress from the interpersonal relationship with the patient and responsibility as an expert rather than the intensity of the work itself has a significant correlation with the mental health state. Further research based on the results of this study will be needed and institutional basis for practical help to reduce occupational stress of dentists.

## Abbreviations

BEPSI-K: Brief Encounter Psychosocial Instrument Korean version
CES-D: Center for epidemiologic studies depression scale
PSQI: Pittsburgh sleep quality index
STAI: State-trait anxiety index

## References

[1] Freeman R, Main JR, Burke FJ (1995). Occupational stress and dentistry: theory and practice. Part I. Recognition. Br Dent J.

[2] Rada RE, Johnson-Leong C (2004). Stress, burnout, anxiety and depression among dentists. Journal of the American Dental Association (1939).

[3] Sancho FM, Ruiz CN (2010). Risk of suicide amongst dentists: myth or reality?. Int Dent J.

[4] de Jonge J, Bosma H, Peter R, Siegrist J (2000). Job strain, effort-reward imbalance and employee well-being: a large-scale cross-sectional study. Social science & medicine (1982).

[5] Kudielka BM, Hanebuth D, von Kanel R, Gander ML, Grande G, Fischer JE (2005). Health-related quality of life measured by the SF12 in working populations: associations with psychosocial work characteristics. J Occup Health Psychol.

[6] Tsutsumi A, Kayaba K, Theorell T, Siegrist J (2001). Association between job stress and depression among Japanese employees threatened by job loss in a comparison between two complementary job-stress models. Scand J Work Environ Health.

[7] Kawakami N, Haratani T, Araki S (1992). Effects of perceived job stress on depressive symptoms in blue-collar workers of an electrical factory in Japan. Scand J Work Environ Health.

[8] Larisch M, Joksimovic L, von Dem Knesebeck O, Starke D, Siegrist J (2003). Effort-reward imbalance at work and depressive symptoms--a cross-sectional investigation of middle-aged employees. Psychother Psychosom Med Psychol.

[9] Ostry A, Maggi S, Tansey J, Dunn J, Hershler R, Chen L, Louie AM, Hertzman C (2007). The impact of psychosocial work conditions on attempted and completed suicide among western Canadian sawmill workers. Scandinavian journal of public health.

[10] Tsutsumi A, Kayaba K, Ojima T, Ishikawa S, Kawakami N (2007). Low control at work and the risk of suicide in Japanese men: a prospective cohort study. Psychother Psychosom.

[11] Almasi K, Belso N, Kapur N, Webb R, Cooper J, Hadley S, Kerfoot M, Dunn G, Sotonyi P, Rihmer Z (2009). Risk factors for suicide in Hungary: a case-control study. BMC psychiatry.

[12] Abbasi M, Nejadsarvari N, Kiani M, Borhani F, Bazmi S, Nazari Tavaokkoli S, Rasouli H (2014). Moral distress in physicians practicing in hospitals affiliated to medical sciences universities. Iran Red Crescent Med J.

[13] Jin DG, Kam S, Kang YS, Cho YK, Lee SW, Kim JY, Ahn SG, Chun BY, Yeh MH. Professional job perception, job stress and job satisfaction of doctors practicing in local clinic in Daegu city. *Korean*. J Prev Med. 2003;36(2):153–62. 25363032

[14] McCue JD (1982). The effects of stress on physicians and their medical practice. N Engl J Med.

[15] Kang MK, Kang YS, Kim JR, Jeong BG, Park KS, Kam S, Hong DY (2007). The levels of psychosocial stress, job stress and related factors of medical doctors practicing at local clinics. Journal of preventive medicine and public health = Yebang Uihakhoe chi.

[16] Newbury-Birch D, Lowry RJ, Kamali F (2002). The changing patterns of drinking, illicit drug use, stress, anxiety and depression in dental students in a UK dental school: a longitudinal study. Br Dent J.

[17] Kay EJ, Lowe JC (2008). A survey of stress levels, self-perceived health and health-related behaviours of UK dental practitioners in 2005. Br Dent J.

[18] Kim JY, Kam S, Kang YS, Cho YK, Lee SW, Jin DG, Ahn SG, Chun BY, Yeh MH (2004). Professional job perception, job stress and job satisfaction of westerm doctors and oriental doctors practicing at local clinic. Journal of preventive medicine and public health=Yebang Uihakhoe chi.

[19] Gale EN (1998). Stress in dentistry. The New York state dental journal.

[20] Desjardins M (1997). Physician suicide. Can something be done?. Canadian family physician Medecin de famille canadien.

[21] Pitts FN, Schuller AB, Rich CL, Pitts AF (1979). Suicide among U.S. women physicians, 1967-1972. Am J Psychiatry.

[22] Simon W (1986). Suicide among physicians: prevention and postvention. Crisis.

[23] Myers HL, Myers LB (2004). 'It's difficult being a dentist': stress and health in the general dental practitioner. Br Dent J.

[24] Alexander RE (2001). Stress-related suicide by dentists and other health care workers. Fact or folklore?. Journal of the American Dental Association (1939).

[25] Moller AT, Spangenberg JJ (1996). Stress and coping amongst south African dentists in private practice. The Journal of the Dental Association of South Africa = Die Tydskrif van die Tandheelkundige Vereniging van Suid-Afrika.

[26] Lang-Runtz H (1984). Stress in dentistry: it can kill you. Journal (Canadian Dental Association).

[27] Kang YS, Kam S, Lee SW, Chun BY, Yeh MH (2001). Job stress and its related factors in south Korean doctors. Korean J Prev Med.

[28] Frank SH, Zyzanski SJ (1988). Psychosocial instrument. The Journal of family practice.

[29] Yim J, Bae J, Choi S, Kim S, Hwang H, Huh B (1996). The validity of modified Korean-translated BEPSI (brief encounter psychosocial instrument) as instrument of stress measurement in outpatient clinic. J Korean Acad Fam Med.

[30] Radloff LS (1977). The CES-D scale: a self-report depression scale for research in the general population. Appl Psychol Meas.

[31] Cho MJ, Kim KH (1998). Use of the Center for Epidemiologic Studies Depression (CES-D) scale in Korea. J Nerv Ment Dis.

[32] Spielberger CD, Gorsuch RL, Lushene RE (1970). Manual for the state-trait anxiety inventory.

[33] Kim J (1978). The relationship of trait anxiety and sociability.

[34] Buysse DJ, Reynolds CF, Monk TH, Berman SR, Kupfer DJ (1989). The Pittsburgh sleep quality index: a new instrument for psychiatric practice and research. Psychiatry Res.

[35] Sohn SI, Kim DH, Lee MY, Cho YW (2012). The reliability and validity of the Korean version of the Pittsburgh sleep quality index. Sleep & breathing = Schlaf & Atmung.

[36] Kim KN, Park JY, Shin TS, Jun KJ, Choi EY, Kim HJ, Lee SH, Yoo TW, Huh BY (1998). Degree of stress and stress-related factors by the Korean version of the BEPSI. J Korean Acad Fam Med.

[37] Jeon HJ, Kim JH, Kim BN, Park SJ, Fava M, Mischoulon D, Kang EH, Roh S, Lee D (2014). Sleep quality, posttraumatic stress, depression, and human errors in train drivers: a population-based nationwide study in South Korea. Sleep.

[38] Hahn D-W, Lee C-H, Chon K-K (1996). Korean adaptation of Spielberger's STAI (K-STAI). Korean J Health Psychol.

[39] Lim HE, Lee MS, Ko YH, Park YM, Joe SH, Kim YK, Han C, Lee HY, Pedersen SS, Denollet J (2011). Assessment of the type D personality construct in the Korean population: a validation study of the Korean DS14. J Korean Med Sci.

[40] Wu H, Liu L, Wang Y, Gao F, Zhao X, Wang L (2013). Factors associated with burnout among Chinese hospital doctors: a cross-sectional study. BMC Public Health.

[41] Wu H, Zhao Y, Wang J-N, Wang L (2010). Factors associated with occupational stress among Chinese doctors: a cross-sectional survey. Int Arch Occup Environ Health.

[42] BMSc NK (2011). Burnout among faculty physicians in an academic health science centre. Health.

[43] Selmanovic S, Ramic E, Brekalo-Lazarevic S, Alic A (2011). Stress at work and burnout syndrome in hospital doctors. Medical archives.

[44] Porto G, Carneiro S, Vasconcelos B, Nascimento M, Leal J (2014). Burnout syndrome in oral and maxillofacial surgeons: a critical analysis. Int J Oral Maxillofac Surg.

[45] Ahola K, Hakanen J (2007). Job strain, burnout, and depressive symptoms: a prospective study among dentists. J Affect Disord.

[46] Gorter RC, Albrecht G, Hoogstraten J, Eijkman MA (1999). Professional burnout among Dutch dentists. Community Dent Oral Epidemiol.

[47] Kulkarni S, Dagli N, Duraiswamy P, Desai H, Vyas H, Baroudi K (2016). Stress and professional burnout among newly graduated dentists. Journal of International Society of Preventive & Community Dentistry.

[48] West CP, Huschka MM, Novotny PJ, Sloan JA, Kolars JC, Habermann TM, Shanafelt TD (2006). Association of perceived medical errors with resident distress and empathy: a prospective longitudinal study. JAMA.

[49] West CP, Tan AD, Habermann TM, Sloan JA, Shanafelt TD (2009). Association of resident fatigue and distress with perceived medical errors. JAMA.

[50] Shanafelt TD, Balch CM, Bechamps G, Russell T, Dyrbye L, Satele D, Collicott P, Novotny PJ, Sloan J, Freischlag J (2010). Burnout and medical errors among American surgeons. Ann Surg.

[51] Fahrenkopf AM, Sectish TC, Barger LK, Sharek PJ, Lewin D, Chiang VW, Edwards S, Wiedermann BL, Landrigan CP (2008). Rates of medication errors among depressed and burnt out residents: prospective cohort study. BMJ (Clinical research ed).

[52] Gorter R, Freeman R, Hammen S, Murtomaa H, Blinkhorn A, Humphris G (2008). Psychological stress and health in undergraduate dental students: fifth year outcomes compared with first year baseline results from five European dental schools. Eur J Dent Educ.

[53] Dunlap JE, Stewart JD (1982). Survey suggests less stress in group offices. Dental economics - oral hygiene.

[54] O'Shea RM, Corah NL, Ayer WA (1984). Sources of dentists' stress. Journal of the American Dental Association (1939).

[55] Jager AJ, Tutty MA, Kao AC (2017). Association between physician burnout and identification with medicine as a calling. Mayo Clin Proc.

[56] Tak HJ, Curlin FA, Yoon JD. Association of Intrinsic Motivating Factors and Markers of physician well-being: a National Physician Survey. J Gen Intern Med. 2017;32(7):739-46. PMID 28168540. 10.1007/s11606-017-3997-y PMC5481224 28168540

